# Palazestrant, a novel oral Complete Estrogen Receptor Antagonist (CERAN) and Selective Estrogen Receptor Degrader (SERD), in patients with ER+/HER2− advanced or metastatic breast cancer: phase 1/2 study results

**DOI:** 10.1186/s13058-025-02049-y

**Published:** 2025-07-01

**Authors:** Erika P. Hamilton, Manish R. Patel, Virginia F. Borges, Jane L. Meisel, Meena Okera, Carlos A. Alemany, Timothy J. Pluard, Robert Wesolowski, Dhanusha Sabanathan, Kathy D. Miller, Alison K. Conlin, Nicole McCarthy, Morena Shaw, Margaret Tonda, Mark Shilkrut, Nancy U. Lin

**Affiliations:** 1 Sarah Cannon Research Institute, 335 24th Ave North Ste 300, Nashville, TN 37203 USA; 2 Florida Cancer Specialists/Sarah Cannon Research Institute, Sarasota, FL USA; 3 Anschutz Medical Campus, University of Colorado Cancer Center, Aurora, CO USA; 4 Winship Cancer Institute, Emory University, Atlanta, GA USA; 5 Cancer Research SA, Adelaide, SA Australia; 6 AdventHealth Cancer Institute, Orlando, FL USA; 7 Saint Luke’s Cancer Institute - Koontz Center for Advanced Breast Cancer, Kansas City, MO USA; 8 Division of Medical Oncology, The Ohio State University Comprehensive Cancer Center, Columbus, OH USA; 9 Faculty of Medicine, Health and Human Sciences, Macquarie University, Sydney, NSW Australia; 10 Indiana University Melvin and Bren Simon Comprehensive Cancer Center, Indianapolis, IN USA; 11 Providence Cancer Institute, Portland, OR USA; 12 ICON Cancer Centre, Auchenflower, QLD Australia; 13 Olema Oncology, San Francisco, CA USA; 14 Department of Medical Oncology, Dana-Farber Cancer Institute, Boston, MA USA

**Keywords:** Complete estrogen receptor antagonist, Selective estrogen receptor degrader, Estrogen receptor-positive human epidermal growth factor receptor 2-negative metastatic breast cancer, Endocrine therapy, Palazestrant, Estrogen receptor mutation

## Abstract

**Background:**

Endocrine resistance is a major challenge in treating patients with ER+ /HER2− metastatic breast cancer (MBC) necessitating a switch from endocrine therapy to more toxic therapies. Mutations in *ESR1* constitute a key mechanism of resistance to endocrine therapy in ER+ /HER2− BC. Therapies that overcome endocrine resistance are needed. Palazestrant is a novel oral complete estrogen receptor (ER) antagonist (CERAN) and selective ER degrader (SERD) belonging to a new class of ER-targeting agents that completely blocks estrogen-induced transcriptional activity, regardless of *ESR1* mutation status. This first-in-human, open-label, multicenter, phase 1/2 dose-escalation/expansion study was designed to determine the recommended phase 2 dose (RP2D) and to evaluate safety, pharmacokinetics, and antitumor activity of palazestrant in patients with ER+ /HER2− MBC with disease progression on prior treatment.

**Methods:**

Adults with ER+ /HER2‒ MBC who received ≥ 1 prior line of endocrine therapy for advanced disease and ≤ 2 prior chemotherapy regimens for metastatic disease were eligible. Patients received once-daily oral palazestrant (30–300 mg) in 28-day cycles until progression or intolerable toxicity.

**Results:**

This study enrolled 146 patients. No dose-limiting toxicities were observed at doses up to 300 mg/day palazestrant. Confirmed partial responses were observed with 60 and 120 mg/day palazestrant. Both doses showed similar and tolerable safety profiles, favorable pharmacokinetics, and steady-state plasma concentrations above the predicted threshold for complete ER inhibition. Greater clinical benefit at palazestrant 120 mg/day (46%) versus 60 mg/day (19%) led to selection of 120 mg/day as RP2D and study expansion dose. At 120 mg/day, the median progression-free survival was 4.8 months (95% CI, 3.5–7.1) overall and 5.6 months (95% CI, 4.8–NE) among patients with cancers with *ESR1* mutations. Most treatment-emergent adverse events (TEAEs) were grade 1–2. The most common TEAEs were nausea (62.8%), vomiting (29.1%), and fatigue (25.6%). The most common grade ≥ 3 TEAE was transient neutropenia (10.5%) managed by dose interruption and reduction.

**Conclusions:**

Palazestrant demonstrated a manageable safety profile, with antitumor activity observed in patients with heavily pretreated cancers with wild-type and *ESR1*-mutated BC. These data support the ongoing phase 3 study evaluating palazestrant in patients with ER+ /HER2 − MBC.

**Trial registration:**

ClinicalTrials.gov, NCT04505826. Registered August 6, 2020.

**Supplementary Information:**

The online version contains supplementary material available at 10.1186/s13058-025-02049-y.

## Introduction

While major advances in breast cancer treatment have been made over the last 20 years, it remains the leading cause of cancer-related death among women worldwide [1]; the most common breast cancer subtype is estrogen receptor-positive (ER+), human epidermal growth factor receptor 2-negative (HER2–) cancer, accounting for approximately 70% of all cases [2–5].

The combination regimen of a cyclin-dependent kinase 4/6 (CDK4/6) inhibitor and endocrine therapy (e.g., an aromatase inhibitor) is the current first-line standard-of-care treatment for patients with metastatic ER+/HER2– breast cancer [6]. However, resistance to endocrine therapy eventually develops in most patients.

Although emerging antibody–drug conjugates may be an option for some patients, most patients with endocrine-resistant disease are typically treated with chemotherapy [6]; however, the efficacy of later-stage chemotherapy is limited and associated with substantial toxicity [7–10]. Thus, extending endocrine therapy benefit by overcoming endocrine resistance, and thereby delaying cytotoxic chemotherapy and its negative impact on patient quality of life [7], are important treatment goals.

A common mechanism underlying endocrine resistance involves mutations in the estrogen receptor 1 gene (*ESR1*), which codes for the nuclear receptor ERα and is associated with endocrine resistance in up to 50% of metastatic breast cancer (MBC) cases [11, 12]. Prolonged treatment with aromatase inhibitors creates evolutionary pressure to acquire mutations in the ligand-binding domain of ERα, [13] thereby disrupting the therapeutic target [14]. Thus, the ability to suppress the activity of both wild-type and *ESR1*-mutated ERs may constitute a particularly effective therapeutic approach, especially in patients with pre-treated ER+/HER2– breast cancer.

**Fig. 1:**
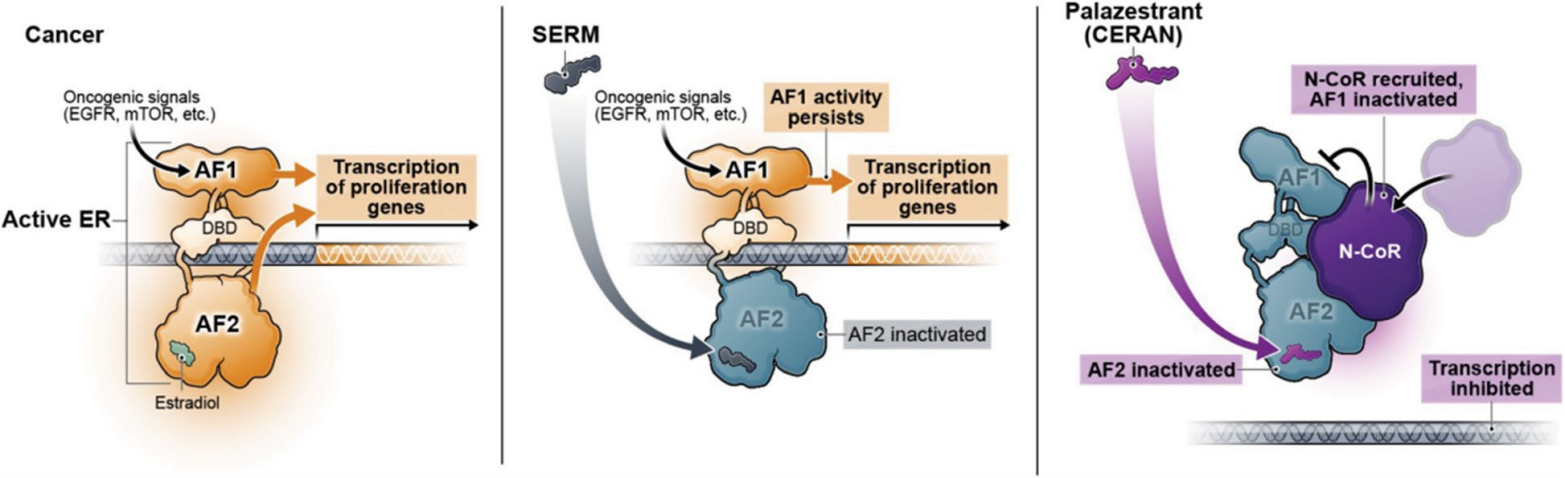
Palazestrant Mechanism of Action. Oncogenic signals activate the ER to drive cancer cell growth. Selective estrogen receptor modulators (SERMs) block only the AF2 domain, leaving the AF1 domain active and capable of gene transcription. Palazestrant is a complete ER antagonist (CERAN) and selective ER degrader (SERD) that acts by blocking both AF1 and AF2 domains to completely block ER-driven transcriptional activity. AF, activation function; DBD, DNA-binding domain; EGFR, epidermal growth factor receptor; ER, estrogen receptor; mTOR, mammalian target of rapamycin; N-CoR, nuclear receptor corepressor, SERM, selective estrogen receptor modulator

Palazestrant (OP-1250) is a novel oral, complete estrogen receptor antagonist (CERAN) and selective ER degrader (SERD) that acts by blocking both transcriptional activation function domains, AF1 and AF2, required for the estradiol-generated transcriptional activity of ER (Fig. 1) [15]. This complete inactivation is reflected in the potent antitumor activity demonstrated by palazestrant in preclinical xenograft models of both *ESR1* wild-type and mutated human breast cancers, where palazestrant treatment resulted in greater tumor shrinkage compared with other endocrine therapies such as fulvestrant and tamoxifen [16–18]. These promising preclinical findings indicate that palazestrant might constitute a more broadly effective endocrine therapy and provide rationale for further clinical development.

Here we present the results of a first-in-human, phase 1/2 study investigating palazestrant for the treatment of advanced or metastatic ER+/HER2− breast cancer.

## Methods

### Study design

**Fig. 2:**
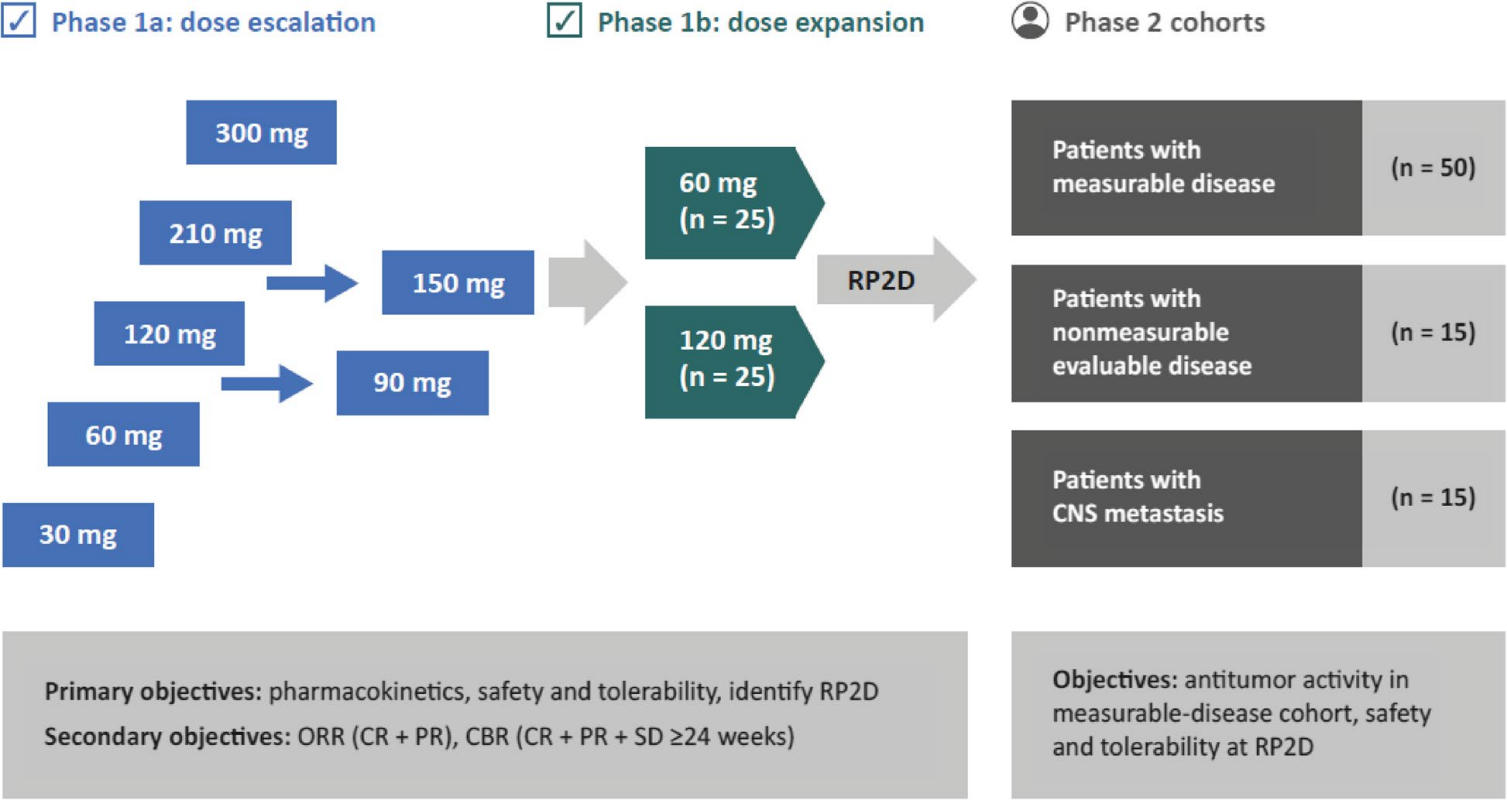
Study design. Phase 1 of the study comprised a dose escalation phase (1a; rolling 6 dose escalation design) to identify the MTD and a dose expansion phase (1b) using selected doses of palazestrant to further characterize the safety, pharmacokinetics, and antitumor activity of treatment and to establish the RP2D. Phase 2 further explored the safety and antitumor activity of palazestrant at the RP2D in patients with measurable disease (primary cohort), nonmeasurable evaluable disease (exploratory cohort), and CNS metastases (exploratory cohort, not included in this report). The data presented in this report include patients from phase 1a/1b as well as cohorts of patients with measurable disease and nonmeasurable evaluable disease from phase 2. CBR, clinical benefit rate; CNS, central nervous system; CR, complete response; MTD, maximum tolerated dose; ORR, overall response rate; PR, partial response; RP2D, recommended phase 2 dose; SD, stable disease

This study (NCT04505826) was an open-label, multicenter, phase 1/2 study designed to identify the maximum tolerated dose (MTD), establish the recommended phase 2 dose (RP2D), and investigate the safety, pharmacokinetics (PK), and antitumor activity of single-agent palazestrant in patients with previously treated ER+/HER2− advanced and metastatic breast cancer (Fig. 2).

Phase 1 of the study comprised a dose-escalation phase (1a) to identify the MTD using a rolling 6 patient dose-escalation design with 7 dose levels. Two doses of palazestrant (60 mg/day and 120 mg/day) were selected for the dose-expansion phase (1b) to further characterize the safety, PK, and preliminary antitumor activity of treatment and to establish the RP2D. Phase 2 further explored the safety and antitumor activity of palazestrant at the RP2D (120 mg/day) in patients with measurable disease (primary cohort), nonmeasurable evaluable disease (exploratory cohort), and central nervous system (CNS) metastases (exploratory cohort).

The data presented here include patients from phase 1a/1b and the measurable disease and nonmeasurable evaluable disease cohorts from phase 2.

### Patient population

Patients eligible for phase 1a of the study included adult men or women of any menopausal status with inoperable histologically or cytologically confirmed ER+/HER2− advanced or metastatic breast cancer who had received at least 1 prior line of endocrine therapy for advanced or metastatic disease for at least 6 months continuously and had received no more than 2 lines of prior chemotherapy regimens for metastatic disease. Pre- or perimenopausal women were required to use luteinizing hormone–releasing hormone agonists for the duration of the study. Patients who had disease progression within the first 2 years from the start of adjuvant endocrine therapy were not eligible, since these patients were considered to have tumors with primary endocrine resistance. Eligible patients had an Eastern Cooperative Oncology Group (ECOG) performance status of 0 or 1 and had evaluable disease. Measurable disease per Response Evaluation Criteria in Solid Tumors, version 1.1 (RECIST v1.1) was not a requirement for the dose escalation phase. Prior CDK4/6 inhibitors, fulvestrant, alpelisib, everolimus, or poly-adenosine diphosphate ribose polymerase inhibitors were allowed if discontinued at least 2 weeks prior to the first dose of palazestrant.

In phase 1b/2 of the study, eligible patients met the same key inclusion criteria defined in phase 1a and were also required to have measurable disease per RECIST v1.1, unless they were enrolled in the nonmeasurable disease cohort of patients with nonmeasurable but evaluable disease. In addition, patients must have received no more than 1 prior chemotherapy regimen for metastatic disease and have received at least 1 (but no more than 4) prior lines of endocrine therapy for advanced or metastatic disease.

Patients were excluded from all phases of the study if they had impaired cardiac function, cerebral vascular disease, or a recent thromboembolic event, an ongoing serious infection or gastrointestinal disorder that could affect absorption of study treatment, a clinically significant history of liver disease consistent with Child–Pugh class B or C, other comorbidities that could affect the patient’s ability to participate in the study, or any condition requiring medications not permitted during the study (e.g., strong CYP3 A4 inhibitors/inducers). Patients who received medications with a known risk of QT interval prolongation or Torsades de Pointes were not permitted to enroll in order to allow comprehensive assessment of QT interval prolongation in the study.

### Study procedures

All patients received oral palazestrant capsules once daily, continuously in 28-day treatment cycles. In this first-in-human study, patients were required to fast for 2 h prior to and 1 h after each palazestrant dose. The initial dose cohort received a dose of palazestrant at 30 mg/day, followed by dose cohorts of 60, 90, 120, 150, 210, and 300 mg/day. Dose escalation cohorts enrolled up to 6 patients at each level; each patient was enrolled in only 1 dose cohort. The dose of palazestrant was escalated from the initial dose and each subsequent dose level provided at least 3 patients completed 1 cycle of treatment without experiencing a dose-limiting toxicity (DLT). In the event of 1 of 3 patients experiencing a DLT at a given dose level, a total of 6 patients would be treated at that dose level, and dose escalation would proceed if no more than 1 DLT was observed after 1 cycle of treatment. In the event of 2 or more patients experiencing DLTs at a given dose level, dose escalation stopped, and this dose level was declared the maximally administered dose. Palazestrant treatment was continued until intolerable toxicity, disease progression, study/treatment withdrawal, or death.

### Endpoints

The MTD was defined as the highest feasible dose in phase 1a in which fewer than 33% of patients (of at least 6 treated) experienced a treatment-related DLT during cycle 1. In this study, DLTs included any treatment-related fatal adverse event (AE); grade ≥ 3 nonhematologic toxicity (including nausea, vomiting, diarrhea not resolving within 3 days with optimal treatment, and fatigue lasting > 7 days); grade 3 neutropenia (with fever and/or infection); grade 3 thrombocytopenia (with bleeding); grade ≥ 4 hematologic toxicity; grade ≥ 2 AEs that resulted in treatment discontinuation or interruption for at least a week (during cycle 1) or a delay of cycle 2 for at least a week; and protocol-defined elevations of aspartate aminotransferase (AST) or alanine aminotransferase (ALT).

The assessment of palazestrant single- and multiple-dose PK parameters included the maximum plasma concentration (C_max_), minimum plasma concentration (C_min_), average plasma concentration (C_avg_), area under the concentration–time curve from 0–24 h (AUC_0–24_), effective half-life (t_1/2_), and time to maximum plasma concentration (T_max_).

Safety assessments included the monitoring of AEs, clinical laboratory parameters, electrocardiograms, and vital signs. AE severity was graded according to the National Cancer Institute Common Terminology Criteria for Adverse Events (version 5.0).

Key efficacy end points included assessment of clinical response according to RECIST v1.1; the clinical benefit rate (CBR), defined as the proportion of patients who achieved a complete response (CR), achieved a partial response (PR), or had stable disease (SD) for at least 24 weeks; and progression-free survival (PFS), defined as the time from the date of the first dose to disease progression or death. Biomarker assessments included changes from baseline in circulating tumor DNA (e.g., *ESR1* variants) and tumor tissue biomarkers (ERα and Ki67).

### Biomarkers

All immunohistochemistry analyses from tumor samples were performed at ICON Central Laboratories (Farmingdale, NY, USA) using the Ventana® BenchMark ULTRA™ automated staining instrument (Oro Valley, AZ, USA). Positive- and negative-control tissue and negative reagent controls were run for each marker, as well as low-ER controls for each run. Estrogen receptor levels were estimated as the number of positive tumor nuclei divided by the total number of tumor nuclei, expressed as a percentage for each receptor type [19]. Evaluation of Ki67 was performed in accordance with Ki67 Working Group recommendations [20, 21]: 1) only nuclear staining was assessed as positive; 2) in cases of tumor heterogeneity (hot spots), 2 counts were performed and the average was reported; and 3) lymphocyte-rich areas were avoided when possible. Presence of activating *ESR1* mutations (amino acid regions 370–381, 460–473, and 529–538) and their mutant allele fractions (MAFs) were determined in cell-free DNA (cfDNA), collected at baseline and at 8 weeks on treatment using the SafeSEQ Breast Cancer Panel (Sysmex Inostics, Baltimore, MD, USA).

### Statistical analysis

All DLT analyses (phase 1a) were performed in patients enrolled in the study during cycle 1 and who either received 75% of the planned palazestrant doses or experienced a DLT during cycle 1. All PK analyses were performed in patients who received at least 1 dose of palazestrant and had adequate postbaseline PK samples for characterization of PK parameters. All safety analyses were performed and PFS assessed in patients who received at least 1 dose of palazestrant. Clinical response (CR or PR) was assessed in patients who received at least 1 cycle of palazestrant, had measurable disease at baseline, and had at least 1 postbaseline tumor assessment. CBR was assessed in patients who received at least 1 cycle of palazestrant, had at least 1 postbaseline tumor assessment, and started their first dose at least 24 weeks before data cutoff. Patients who discontinued study treatment without radiographic progression within 24 weeks from the first dose were not included in the CBR analysis. PK parameters were estimated using conventional noncompartmental methods. For CBR, estimates were accompanied by 2-sided 95% confidence intervals (95% CIs). For PFS, Kaplan–Meier analyses were used to determine medians, event-free rates, and 95% CIs. For all other outcomes, categorical data were analyzed using frequencies and percentages, and continuous data were analyzed using means (standard deviation) or medians (range), as appropriate.

A sample size of approximately 30 patients per dose group in the phase 1b dose expansion cohorts was expected to provide an estimated precision of ± 19% for any binary event rate assessment. In phase 2, a sample size of approximately 50 patients treated at the RP2D was expected to provide an estimated precision of ± 15% for binary assessments.

## Results

The analysis presented here includes clinical and laboratory data from 146 patients recruited at 15 sites in the US and Australia between August 2020 and November 2022.

### Dose escalation and dose selection

During the dose escalation phase of the study, 42 patients were treated with once-daily oral palazestrant at doses ranging from 30 to 300 mg (Fig. 2). Baseline demographics and characteristics of patients in the dose-escalation cohorts are presented in Supplemental Table 1. Palazestrant doses of up to 300 mg/day resulted in no DLTs, and the MTD was not reached. The majority of treatment-emergent adverse events (TEAEs) were grade 1 or 2 in severity in all dose-escalation cohorts of palazestrant (Supplemental Table 2). Indications of antitumor activity were observed in nearly all cohorts, including a confirmed PR in the 60 mg/day and 120 mg/day dose cohorts (Supplemental Fig. 1).

**Fig. 3:**
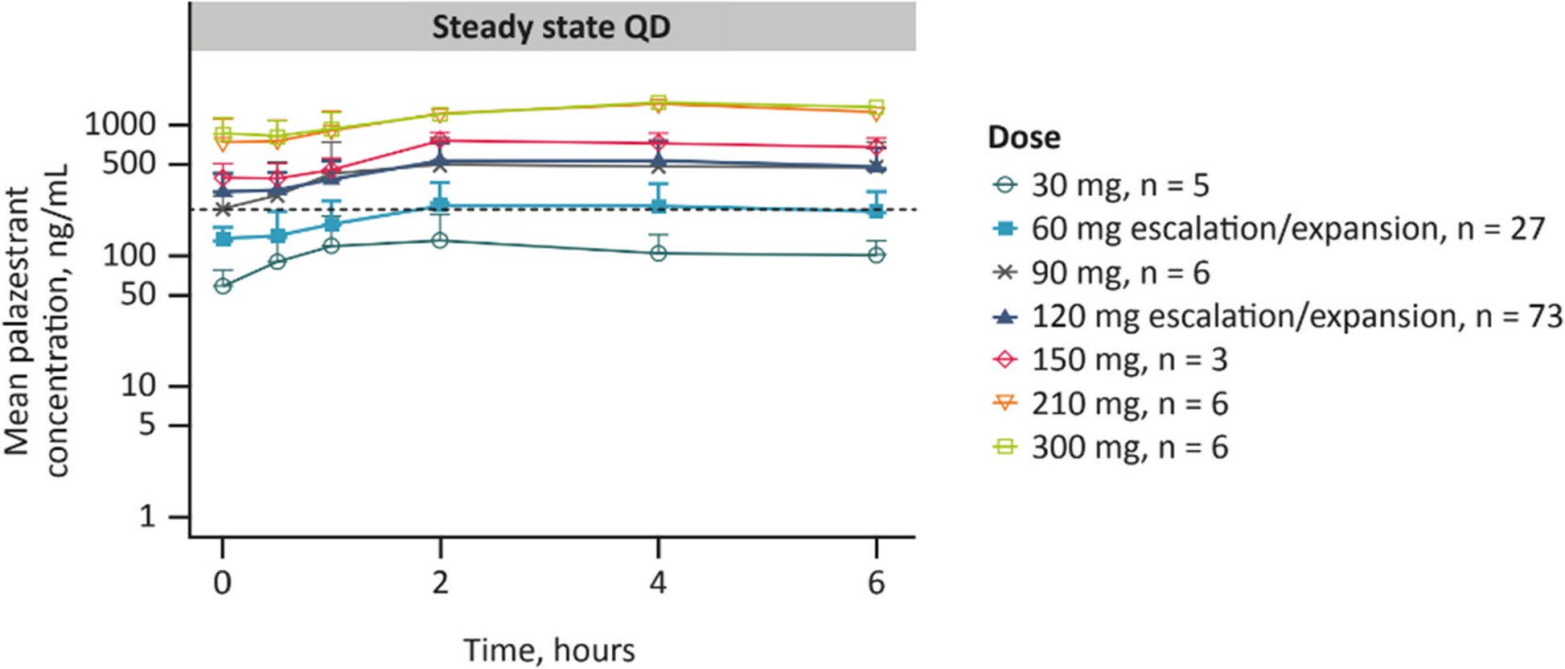
Steady-State Plasma Concentration–Time Profiles for Palazestrant (30 to 300 mg QD; Phase 1a/1b/2 data). Palazestrant was administered once daily at the indicated dose. Plasma samples were collected on cycle 2, day 1, at the indicated timepoints following administration to determine plasma drug concentration. Data presented are arithmetic mean and standard deviation; the heavy dashed black line represents the target (efficacious) exposure based on estradiol-supplemented preclinical models (C_min_: 226 ng/mL). C_min_, minimum plasma concentration; QD, once daily

At the end of the dose-escalation phase, a comprehensive review of safety, efficacy, and PK data was conducted, and both the 60 mg/day and 120 mg/day doses were selected for further study in the dose-expansion phase (phase 1b). Dose selection was based on the observation of confirmed PRs at both dose levels (Supplemental Fig. 1), tolerability (Supplemental Table 2), and PK data indicating that palazestrant steady-state plasma concentrations reached the predicted efficacy threshold based on estradiol-supplemented preclinical models for both doses (Fig. 3) [22].

### Patient population

Here we have summarized data for the 60 mg/day and 120 mg/day dose levels, given that these doses were selected for further exploration in dose expansion cohorts (phase 1b), and 120 mg/day was further defined to be the RP2D.

Baseline demographics and characteristics for all patients treated at 60 mg/day or 120 mg/day (*n* = 116; including those from the dose-escalation phase) are presented in Table 1. Patients had a median age of 61 years, the majority had measurable or visceral disease (84.5% and 73.3%, respectively), 74.1% of patients received at least 2 prior lines of systemic therapy in the advanced setting (median 2; range, 1–8), approximately two-thirds (63.8%) received at least 2 prior lines of endocrine therapy in the advanced setting (median 2; range, 1–5), and all but 5 patients (95.7%) received prior CDK4/6 inhibitor therapy. Approximately half of all patients with evaluable circulating tumor DNA (ctDNA) at baseline had *ESR1* mutations (51/96 [53.1%]).

**Table 1:** Baseline patient demographics and characteristics

Characteristic	Palazestrant, 60 mg (*n* = 30)	Palazestrant, 120 mg (*n* = 86)	Total (*N* = 116)
Age, years; median (range)	59.5 (30–81)	61.0 (32–85)	61.0 (30–85)
Female, n (%)	30 (100)	86 (100)	116 (100)
Race, n (%)			
White	23 (76.7)	70 (81.4)	93 (80.2)
Black or African American	1 (3.3)	7 (8.1)	8 (6.9)
Asian	1 (3.3)	3 (3.5)	4 (3.4)
Not reported/Other	5 (16.7)	6 (7.0)	11 (9.5)
Childbearing potential,^a^ n (%)			
Yes	0	7 (8.1)	7 (6.0)
No	30 (100)	79 (91.9)	109 (94.0)
ECOG performance status, n (%)			
0	19 (63.3)	46 (53.5)	65 (56.0)
1	11 (36.7)	40 (46.5)	51 (44.0)
Measurable disease, n (%)	29 (96.7)	69 (80.2)	98 (84.5)
Visceral disease,^b^ n (%)	24 (80.0)	61 (70.9)	85 (73.3)
Prior lines of therapy in advanced setting, n (%)			
1	9 (30)	21 (24.4)	30 (25.9)
2	6 (20.0)	29 (33.7)	35 (30.2)
3	6 (20.0)	19 (22.1)	25 (21.6)
4	6 (20.0)	10 (11.6)	16 (13.8)
≥ 5	3 (10.0)	7 (8.1)	10 (8.6)
Prior lines of endocrine therapy in advanced setting,^c^ n (%)			
1	12 (40.0)	30 (34.9)	42 (36.2)
2	7 (23.3)	32 (37.2)	39 (33.6)
3	7 (23.3)	15 (17.4)	22 (19.0)
4	3 (10.0)	8 (9.3)	11 (9.5)
5	1 (3.3)	1 (1.2)	2 (1.7)
Type of prior therapy in advanced setting, n (%)			
CDK4/6 inhibitor	28 (93.3)	83 (96.5)	111 (95.7)
Aromatase inhibitor	23 (76.7)	73 (84.9)	96 (82.8)
Fulvestrant	19 (63.3)	57 (66.3)	76 (65.5)
Chemotherapy	12 (40.0)	27 (31.4)	39 (33.6)
*ESR1* mutations (ctDNA) at baseline, n/N (%)	15/21 (71.4)	36/75 (48.0)	51/96 (53.1)

*CDK4/6* cyclin-dependent kinase 4/6, *ctDNA* circulating tumor DNA, *ECOG* Eastern Cooperative Oncology Group, *ESR1* estrogen receptor 1 gene

^a^The reproductive status of female participants has been reported as having a childbearing potential (yes vs no), with “yes” corresponding to pre- and perimenopausal women

^b^Liver, lung, peritoneum, pleura, ascites

^c^Endocrine therapies included aromatase inhibitors, fulvestrant, investigational endocrine therapy, and selective estrogen receptor modulators

Among all patients treated with 60 or 120 mg/day palazestrant, 102/116 (87.9%) had discontinued treatment at the time of data cutoff (July 7, 2023) and 14 (12.1%) were ongoing. Reasons for treatment discontinuation included radiographic disease progression (*n* = 78 [67.2%]), clinical disease progression (i.e., without disease progression per RECIST v1.1) (*n* = 11 [9.5%]), AEs (*n* = 7 [6.0%]), withdrawal of consent (*n* = 4 [3.4%]), and other reasons (*n* = 2 [1.7%]).

### Safety

Overall, the incidence of the most common AEs for the individual palazestrant dose cohorts (60 mg/day and 120 mg/day) were similar (Table 2). Most patients (95.3%) who received the RP2D of 120 mg/day experienced ≥ 1 TEAE of any grade, most commonly nausea (62.8%), vomiting (29.1%), and fatigue (25.6%). The majority of TEAEs were grade 1 or 2 in severity. Grade 3 TEAEs were reported in 21 (24.4%) patients, most commonly vomiting (*n* = 4 [4.7%]), neutropenia, nausea, and fatigue (*n* = 3 each [3.5%]). Eight (9.3%) patients treated with 120 mg/day palazestrant also experienced grade 4 TEAEs, including neutropenia (*n* = 6), acute respiratory failure (*n* = 1), and 1 patient experienced 3 grade 4 events (pulmonary embolism, diverticulitis, and acute left ventricular failure).

**Table 2:** Adverse Events Occurring in ≥ 15% of Patients Overall^a^

	**Palazestrant, 60 mg (*n* = 30)**			**Palazestrant, 120 mg (*n* = 86)**		
	**Any grade**	**Grade 3**	**Grade 4**	**Any grade**	**Grade 3**	**Grade 4**
TEAE, n (%)						
Nausea	14 (46.7)	0	0	54 (62.8)	3 (3.5)	0
Vomiting	5 (16.7)	0	0	25 (29.1)	4 (4.7)	0
Fatigue	13 (43.3)	0	0	22 (25.6)	3 (3.5)	0
Neutropenia^a,b^	2 (6.7)	0	0	21 (24.4)	3 (3.5)	6 (7.0)
Headache	7 (23.3)	0	0	17 (19.8)	0	0
Constipation	6 (20.0)	0	0	15 (17.4)	0	0
Diarrhea	6 (20.0)	1 (3.3)	0	12 (14.0)	0	0
TRAE, n (%)						
Nausea	10 (33.3)	0	0	47 (54.7)	3 (3.5)	0
Vomiting	3 (10.0)	0	0	21 (24.4)	2 (2.3)	0
Fatigue	9 (30.0)	0	0	17 (19.8)	2 (2.3)	0
Neutropenia^a,b^	0	0	0	18 (20.9)	3 (3.5)	5 (5.8)
Headache	3 (10.0)	0	0	13 (15.1)	0	0

*AE* adverse event, *TEAE* treatment-emergent adverse event, *TRAE* treatment-related adverse event

^a^Patients could report more than 1 type of AE, but at each level of summation patients are included only once

^b^Includes preferred terms neutropenia, neutrophil count decreased, and febrile neutropenia

The majority of treatment-related adverse events (TRAEs) were grade 1 or 2 in severity (Table 2). The most common TRAEs of any grade were nausea (54.7%), vomiting (24.4%), fatigue (19.8%), neutropenia (20.9%), and headache (15.1%). Grade 3 treatment-related events occurred in 10 (11.6%) patients. Nausea and neutropenia (*n* = 3 each [3.5%]) and vomiting and fatigue (*n* = 2 each [2.3%]) were the most common grade 3 TRAEs. Treatment-related grade 4 neutropenia occurred in 5 (5.8%) patients.

Seven patients discontinued palazestrant due to AEs, of which 5 were assessed as related to palazestrant: grade 4 febrile neutropenia with no evidence of infection, grade 4 neutropenia, grade 3 nausea, grade 3 international normalized ratio increase, and grade 2 fatigue. Of the 6 patients with grade 4 neutropenia, 3 events resolved with a dose interruption (1 week) followed by a dose reduction (to 60 mg/day in 1 patient and to 90 mg/day in 2 patients); these patients continued treatment with no further neutropenia. In 3 patients, grade 4 neutropenia resolved following discontinuation of palazestrant. Grade 4 neutropenia occurred in 1 patient, concurrent with disease progression, and was assessed as not related to palazestrant.

There were no clinically significant changes in the QT interval corrected by the Fridericia formula (QTcF) in any patients treated with palazestrant, and no QTcF values exceeded 500 ms. “Class-effect” TEAEs of oral SERDs observed in less than 10% of patients treated at the RP2D of palazestrant included bradycardia (9%) and photopsia (5%); all bradycardia and photopsia events were grade 1, and there were no dose modifications, interruptions, or discontinuations due to these events.

### Pharmacokinetics

Single-dose and steady-state PK analyses (cycles 1 and 2, day 1) showed that palazestrant was readily bioavailable and demonstrated rapid absorption with a median T_max_ of approximately 4 h (Table 3). Palazestrant also showed linear PK with a dose-proportional exposure in the dose range of 30 mg/day to 300 mg/day. Steady-state PK profiles showed limited peak-to-trough fluctuation (range, 1.89–2.51), with an accumulation ratio of approximately 3 for C_max_ (range, 1.96–3.55) and of approximately 4 for AUC_0–24_ (range, 3.63–5.20), and an effective half-life of approximately 70 h (range, 50.5 h to 77.2 h). The steady*-*state geometric mean AUC_0–24_ was 4030 and 9210 h*ng/mL for 60 and 120 mg dose cohorts, respectively (Table 3). Steady-state plasma concentration profiles showed that doses of ≥ 60 mg/day resulted in palazestrant concentrations above the predicted efficacy threshold of 226 ng/mL (Fig. 3), indicating complete inhibition of the ER. At the RP2D of 120 mg (*n* = 73), the geometric mean C_max_ at steady state was 541 ng/mL, and the geometric mean C_avg_ was 384 ng/mL, showing sustained concentrations above the predicted efficacy threshold (Table 3). Overall, palazestrant showed biphasic elimination in the distribution phase, which was similar to that observed with 120 mg palazestrant in healthy volunteers (data on file), confirming the 8-day terminal half-life.

**Table 3:** Steady-State PK Parameters for Palazestrant^a^

Dose QD	T_max,ss_ (h)	C_min,ss_ (ng/mL)	C_max,ss_ (ng/mL)	C_avg,ss_ (ng/mL)	AUC_0–6,ss_ (ng*h/mL)	AUC_0–24,ss_ (ng*h/mL)^b^	AR C_max,ss_^c^	AR AUC_0–24,ss_^d^	PTR^e^
30 mg (*n* = 5)	2.0 [1.0–6.0]	55.4 (32.5)	139 (49.6)	82.0 (32.7)	597 (51.2)	1970 (32.7)	2.02 (16.7)	3.83 (22.3)	2.51 (23.9)
**60 mg** **(*n* = 27)**	**4.0** **[1.00–6.00]**	**116 (50.3)**	**241 (47.1)**	**168 (45.2)**	**1160 (48.3)**^**f**^	**4030** **(45.2)**^**f**^	**2.25 (57.5)**	**2.94 (51.0)**^**g**^	**2.07 (23.5)**
90 mg (*n* = 6)	4.0 [1.00–6.00]	213 (92.7)	460 (77.4)	322 (82.1)	2250 (86.2)^f^	7720 (82.1)^f^	3.55 (34.7)^g^	5.12 (41.3)^g^	2.16 (21.9)
**120 mg** **(*n* = 73)**	**4.0** **[1.00–6.00]**	**276 (41.6)**	**541 (37.4)**	**384 (37.9)**	**2690 (37.0)**^**f**^	**9210** **(37.9)**^**f**^	**1.96 (19.6)**^**g**^	**4.76 (43.1)**^**g**^	**1.96 (19.6)**
150 mg (*n* = 3)	2.0 [2.00–4.00]	370 (32.4)	789 (11.1)	549 (20.3)	3880 (16.7)	13,200 (20.3)	2.33 (27.3)	4.24 (20.7)	2.13 (24.4)
210 mg (*n* = 6)	4.0 [2.00–4.00]	660 (49.3)	1450 (39.5)	941 (47.3)	6780 (40.6)	22,600 (47.3)	3.30 (47.4)	5.20 (39.7)	2.20 (20.7)
300 mg (*n* = 6)	4.0 [2.00–6.00]	744 (36.5)	1410 (47.0)	1060 (40.3)	7000 (41.9)	25,500 (40.3)	2.13 (55.1)	3.63 (49.8)	1.89 (16.1)

*AR* accumulated ratio, *AUC* area under the concentration–time curve, *AUC*_*0–6,ss*_ area under the concentration–time curve from time 0 to 6 h post dose at steady state, *AUC*_*0–24*_ area under the concentration–time curve from time 0 to 24 h post dose, *AUC*_*0–24,ss*_ area under the concentration–time curve from time 0 to 24 h post dose at steady state, *C*_*avg,ss*_ average plasma concentration at steady state, *C*_*max*_ maximum plasma concentration, *C*_*max,ss*_ maximum plasma concentration at steady state, *C*_*min,ss*_ minimum plasma concentration at steady state, *GeoMean* geometric mean, *GeoCV* geometric coefficient of variation, *PK* pharmacokinetics, *PTR* peak-to-trough ratio, *QD* once daily, *t*_*1/2*_ effective half life, *T*_*max*_ time to maximum plasma concentration, *T*_*max,ss*_ time to maximum plasma concentration at steady state

^a^GeoMean (GeoCV%) data are presented for all parameters, except for T_max_, where median (min–max) data are presented. Data from May 2023 data cut. Rows indicated by bold font/shading represent doses selected for further evaluation in the dose expansion cohort and the recommended phase 2 dose

^b^24-hour palazestrant imputed using the observed predose value for computation of AUC_0–24,ss_

^c^AR C_max,ss_ = C_max_ on cycle 2, day 1/C_max_ on cycle 1, day 1

^d^AR AUC_0–24,ss_ = AUC_0–24_ on cycle 2, day 1/AUC_0–24_ on cycle 1, day 1

^e^PTR = C_max,ss_/C_trough,ss_

^f^Some patients had insufficient sampling data to calculate AUC or t_½_

^g^A lower patient count compared with the total number of patients in the dose cohort was calculated due to missing data needed to calculate AR

### Efficacy

Of the 116 patients treated in the 60-mg and 120-mg dose cohorts, 92 (79.3%) patients with measurable disease were evaluable for tumor response.

Antitumor activity was observed in patients (*n* = 26) with measurable disease receiving 60 mg/day, with 2 confirmed PRs (cPRs; 7.7%) and 1 unconfirmed PR (3.8%) for a total of 3 PRs (11.5%). The CBR was 19.2% (95% CI, 6.6–39.4) in all evaluable patients who received 60 mg/day palazestrant (*n* = 26) and 23.1% (95% CI, 5.0–53.8) among the 13 patients with cancers with *ESR1* mutations. More patients stayed on treatment longer in the 120 mg dose cohort vs the 60 mg cohort, and CBR among patients treated with 120 mg was 45.7% compared to 19.2% in those treated with 60 mg.

**Fig. 4:**
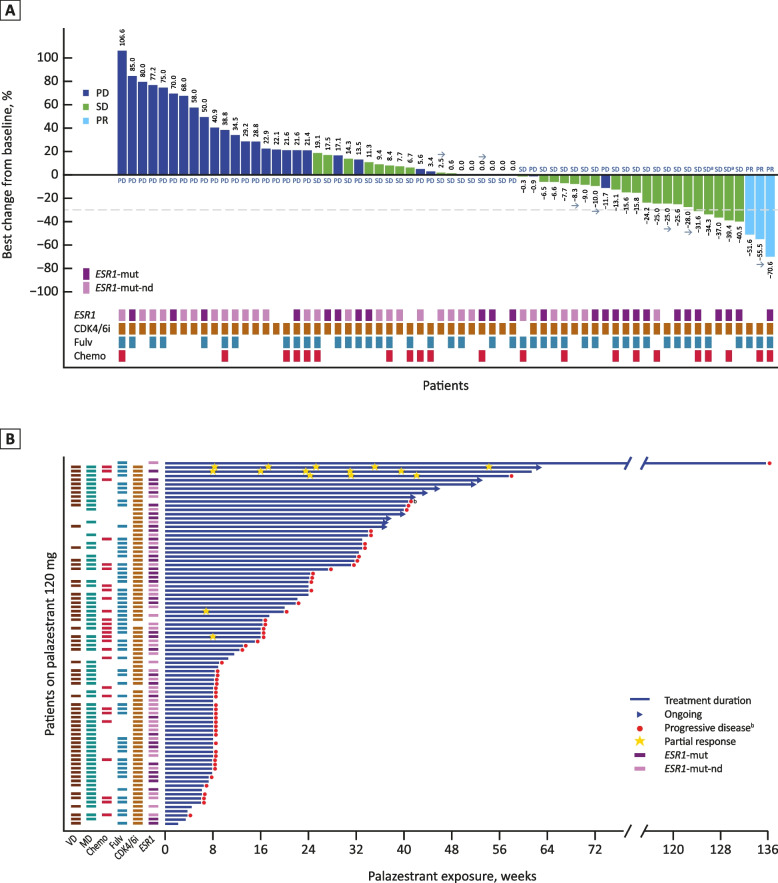
Antitumor Activity in Patients Receiving 120 mg/day Palazestrant. **A** Best overall response and change from baseline in target lesion size among the 64 patients with evaluable baseline and postbaseline target lesions (66 response-evaluable patients treated with 120 mg/day palazestrant; 2 patients did not have evaluable postbaseline target lesions). **B** Duration of treatment and best overall responses among patients receiving 120 mg/day palazestrant (*n* = 86). Arrows represent patients still receiving treatment; the absence of a box represents missing data. All prior treatments are in the advanced disease setting. In (**B**), each lane represents 1 patient. Reasons for treatment discontinuation included disease progression, adverse events, withdrawal of consent, and other reasons (treatment was discontinued in 1 patient after a post-treatment biopsy showed HER2-positive disease; 1 patient discontinued treatment for financial reasons). ^a^Unconfirmed PRs. ^b^One patient continued treatment beyond progression due to clinical benefit per principal investigator assessment. CDK4/6i, cyclin-dependent kinase 4/6 inhibitor; chemo, chemotherapy; *ESR1*, estrogen receptor 1 gene; *ESR1*-mut, *ESR1*-mutation detected; *ESR1*-mut-nd, ESR1 mutation not detected; fulv, fulvestrant; HER2, human epidermal growth factor receptor 2; MD, measurable disease; PD, progressive disease; PR, partial response; SD, stable disease; VD, visceral disease

**Fig. 5:**
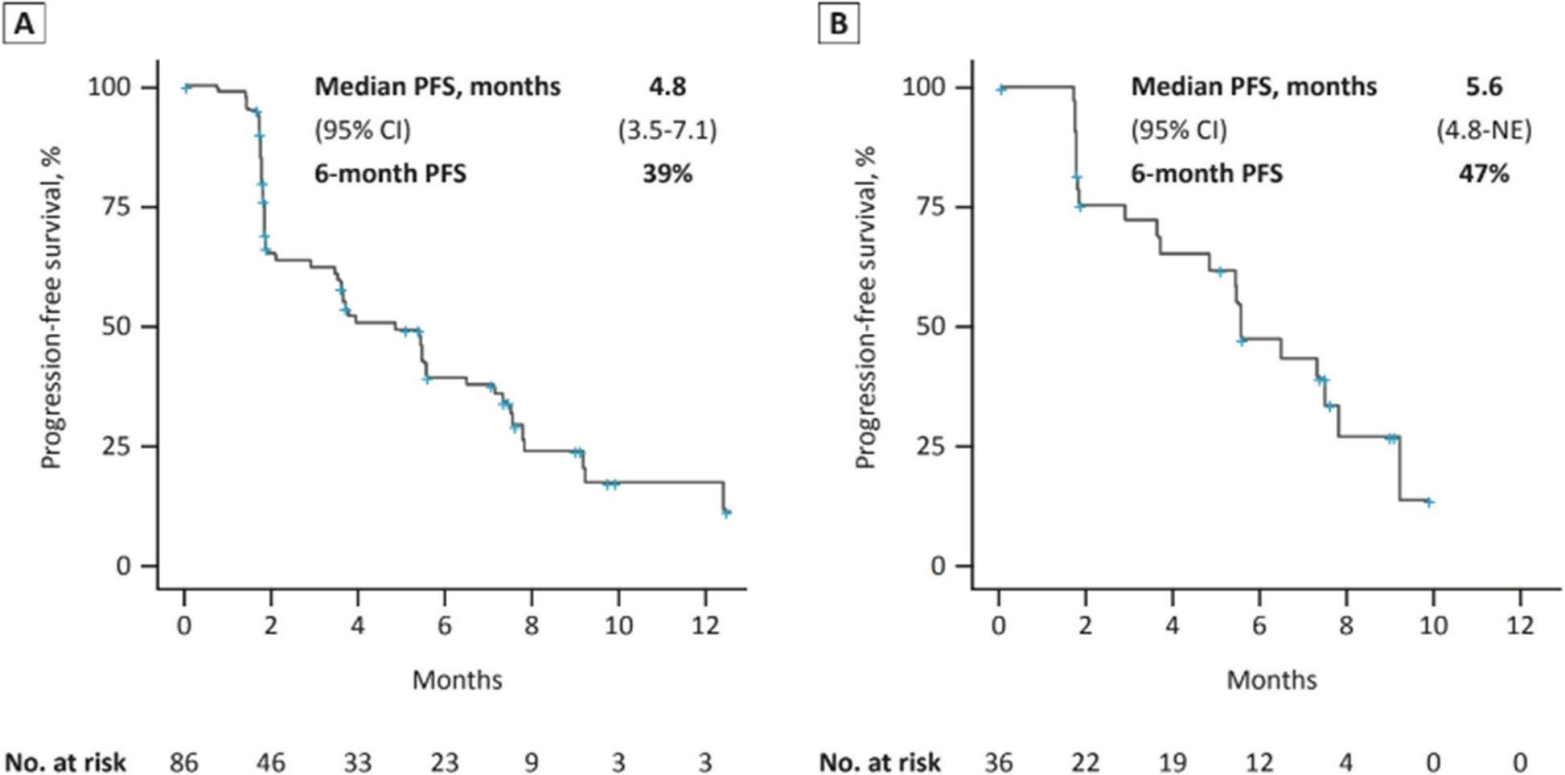
Progression-free survival. Kaplan–Meier survival curves for progression-free survival. **A** All patients receiving 120 mg/day palazestrant (*n* = 86). **B** Patients receiving 120 mg/day palazestrant who had an *ESR1* mutation at baseline (*n* = 36). *ESR1*, estrogen receptor 1 gene; NE, not estimable; PFS, progression-free survival

Among the 66 patients (56.9% of the total population) with measurable disease treated at the RP2D, there were 3 confirmed PRs (4.5%) and 2 unconfirmed PRs (3.0%) for a total of 5 PRs (7.6%). One additional patient treated at the RP2D had a cPR after the data cutoff date for a total of 4 confirmed PRs (6.0%). The CBR was 45.7% (95% CI, 33.7–58.1) in all evaluable patients who received 120 mg/day palazestrant (*n* = 70) and 58.6% (95% CI, 38.9–76.5) in patients with cancers with *ESR1* mutations (*n* = 29). A reduction in tumor size (percentage change less than zero) was experienced by 25/64 (39.1%) patients treated at the R2PD, including 8/64 (12.5%) who experienced a > 30% reduction (Fig. 4A). Palazestrant treatment led to prolonged disease stabilization, with 32/86 (37.2%) of patients treated at 120 mg/day remaining on treatment for ≥ 6 months and 6 (7.0%) remaining on treatment for ≥ 1 year (Fig. 4B). The median PFS for patients receiving 120 mg/day palazestrant was 4.8 months (95% CI, 3.5–7.1), with a 6-month PFS rate of 39% (Fig. 5A). In patients with cancers with *ESR1* mutations treated at the R2PD, the median PFS was 5.6 months (95% CI, 4.8-NE), and the 6-month PFS rate was 47% (Fig. 5B).

### Biomarker analyses

In patients who underwent biomarker testing, samples collected on cycle 3 day 1 of treatment across all dose levels were compared to baseline samples to assess changes during treatment. Palazestrant treatment was associated with significantly reduced Ki67 levels from 29.2% (*N* = 18) to 20.4% (*N* = 12); *P* = 0.041 (Supplemental Fig. 2 A) and resulted in a numerical decrease in ERα Allred score from 6.67 (*N* = 18) to 5.17 (*N* = 12); *P* = 0.13 (Supplemental Fig. 2B). Additionally, the number of endocrine resistance-associated *ESR1*-variant mutations (Y537S, Y537 N/C, L536H/P/R, and D538G) [23] were reduced from 106 mutations detected at baseline (in 64 patients with detectable mutations at baseline) to 37 mutations (in 21 patients) after 2 treatment cycles of palazestrant treatment (Supplemental Fig. 2 C). In patients who had been treated with the RP2D of 120 mg/day palazestrant and had ctDNA available for analysis, the CBR was 73% (8/11) in patients who had a reduction in *ESR1* mutations and 25% (8/32) in those who did not have a reduction in *ESR1* mutations.

## Discussion

The results from this first-in-human, phase 1/2 study showed that once-daily oral palazestrant, a novel oral CERAN, at doses of 30 mg/day to 300 mg/day had no DLTs, and no MTD was identified. Based on the tolerable safety profile, favorable PK, and encouraging antitumor activity shown in the phase 1 portion of the study, the 120 mg/day dose was chosen as the RP2D. Furthermore, because patients stayed on this treatment longer, the CBR was numerically better, and the safety profile was similar to that of the 60 mg dose, this dose was further explored in phase 2 of the study. Limited data were available for patients treated with 90 mg palazestrant, as only 6 patients were enrolled during dose escalation. A phase 3 study OPERA-01 (NCT06016738) is ongoing where two doses of 90 or 120 mg/day palazestrant are being investigated in the dose optimization part. The optimal dose of palazestrant to be used in the rest of OPERA-01 study (90 mg/day or 120 mg/day) will be selected based on safety, efficacy, PK profile and patient-reported outcomes.

PK analyses showed that palazestrant was readily bioavailable and rapidly absorbed (reaching peak concentration after 4 h), with extensive tissue distribution, dose-proportional exposure, and a long half-life of approximately 8 days. These PK characteristics support once-daily dosing.

Palazestrant was well tolerated at the RP2D of 120 mg/day, with the majority of TRAEs being transient, tolerable, and grade 1 or 2 in severity. The most frequently reported TRAEs were gastrointestinal events and fatigue, which have also been commonly reported with SERDs and selective ER covalent antagonists (SERCAs) [24–27]. In this study, palazestrant was administered in the fasted state, proton pump inhibitors and most common antiemetic medications were not permitted, and the drug was administered in a relatively large capsule formulation, each of which could have contributed to an increased incidence of gastrointestinal events. Ongoing studies are using a tablet formulation, allowing drug administration in the fed state, and using anti-emetic medications when needed that may reduce frequency of nausea, vomiting, and fatigue [26]. In patients treated with the RP2D of palazestrant, the rate of treatment-related diarrhea of any grade was 13%, and there were no grade ≥ 3 events. In contrast, rates of dose-limiting diarrhea have been reported in up to 33% of patients following treatment with some SERDs [27]. Additionally, unlike with other SERDs that have reported rates of up to 45% for bradycardia and 56% for photopsia, the incidence of bradycardia and photopsia was lower with palazestrant (9% and 5%, respectively) [25, 28, 29].

Treatment-related grade 4 neutropenia was observed in 5 patients (6%) who were administered palazestrant at 120 mg/day. Events of neutropenia are uncommon with endocrine monotherapy [30–33]. While grade 4 neutropenia events occurred approximately 6-8 weeks after initiation of palazestrant, these were managed with dose interruptions or discontinuations and/or with colony-stimulating factors, and following dose reductions, 3 patients were able to continue palazestrant with no further recurrences of neutropenia. In the other 2 patients, neutropenia resolved upon treatment discontinuation. The mechanism underlying neutropenia is unknown, and no corresponding trends were observed in preclinical studies of palazestrant (unpublished data). Research is ongoing to investigate etiology and predisposing factors for neutropenia. All ongoing studies with palazestrant include proactive monitoring of blood counts that is more frequent during first 6–8 weeks after initiation of palazestrant, with the possibility for dose reduction or interruption following the detection of grade 3 or 4 neutropenia. Neutropenia, albeit at a lower frequency, has also been reported with fulvestrant, with grade ≥ 3 neutropenia observed in 1% to 2% of patients receiving fulvestrant monotherapy and rates of febrile neutropenia of 0 to 1% [30, 31]. Whether neutropenia is a result of the mechanism of action of palazestrant, prior anticancer treatments, or the underlying disease remains to be elucidated. Although neutropenia was observed with palazestrant monotherapy in this study, results from 2 separate phase 1b/2 studies showed that combination of full-dose palazestrant with palbociclib [34] or with ribociclib [35, 36] was feasible in patients with advanced or metastatic ER+/HER2− breast cancer. The incidence and severity of AEs were consistent with those of palbociclib or ribociclib, with no increase of neutropenia in combination regimens [34–40].

The RP2D of 120 mg/day palazestrant demonstrated evidence of antitumor activity, with a CBR of 45.7% and a median PFS of 4.8 months in the overall population and a CBR of 58.6% and a median PFS of 5.6 months among patients with cancers with *ESR1* mutations at baseline. In an analysis of 49 patients who received palazestrant as second- or third-line therapy with or without prior chemotherapy, the median PFS was extended to 7.2 months, with a CBR of 48% overall. Similarly, patients with cancers with *ESR1* mutations had an extended PFS of 7.3 months and CBR of 59% in this subanalysis [41]. While between-study comparisons should always be made with caution because of variations in patient populations, equivalent dosing levels, and study designs, the antitumor activity of palazestrant [41] compares favorably to that observed with the recently approved SERD elacestrant in the EMERALD trial of patients with disease progression on 1 or 2 lines of endocrine therapy and a CDK4/6 inhibitor and ≤ 1 line of prior chemotherapy for advanced disease [33].

Results from this study indicate that novel anti-estrogen agents may have activity in ER+/HER2– breast cancers that showed prior sensitivity to ET. The study did not include patients with primary endocrine resistance. The current consensus is that patients with cancers demonstrating primary endocrine resistance should be offered chemotherapy-based regimens [42]. Furthermore, clinical data suggest that palazestrant may have an advantage over other endocrine therapies in that it completely inactivates the estrogen receptor with its unique mechanism of action as a CERAN and SERD, although a direct comparison has not been performed. Promising antitumor activity was observed in patients regardless of *ESR1* mutation status, suggesting that palazestrant counteracts endocrine resistance driven by *ESR1* mutations. Additionally, the decrease in *ESR1* mutation variants following treatment suggests that palazestrant has activity across clinically relevant *ESR1* variants. Taken together, the decreased instances of *ESR1* mutations associated with endocrine resistance following treatment and the observed antitumor activity in patients with *ESR1*–wild-type disease suggest that palazestrant may suppress the emergence of *ESR1* mutations. While the correlation between reductions in *ESR1* mutations and CBR is encouraging based on a small number of patients, these findings should be further explored in larger trials.

The CERAN/SERD mechanism of action of palazestrant is shared by fulvestrant, camizestrant and imlunestrant [15, 28, 43]. Recent studies showed that imlunestrant may be beneficial only for cancers harboring *ESR1* mutations [43]. Elacestrant, which is a selective ER modulator (SERM) and SERD, does not completely shut down ER signaling due to being an partial agonist and was also more active in cancers harboring *ESR1* mutations [15, 44–46]. Palazestrant demonstrated greater potency in vitro relative to imlunestrant and showed superior pharmacokinetics and preclinical antitumor activity relative to fulvestrant and elacestrant, regardless of *ESR1* mutation status [15].

Limitations of this study, as with most early-phase clinical trials, include its open-label design and lack of a comparator arm that limit the interpretation of the observed safety profile and antitumor activity.

## Conclusions

In this first-in-human phase 1/2 study, palazestrant demonstrated a manageable safety profile with antitumor activity observed in heavily pretreated patients with both wild-type and *ESR1*-mutated breast cancer. At the RP2D of 120 mg/day, palazestrant was well tolerated, with most TEAEs being grade 1 or 2 in severity, and with an overall CBR of 45.7%. The median PFS was 4.8 months (95% CI, 3.5–7.1) overall and 5.6 months (95% CI, 4.8–NE) among patients with cancers with *ESR1* mutations.

The findings from this study support the continued development of palazestrant for the treatment of ER+/HER2− MBC. Palazestrant could result in prolongation of time on endocrine therapy, thereby increasing time to disease progression and delaying the transition to cytotoxic chemotherapy. The ongoing phase 3 study OPERA-01 is evaluating palazestrant monotherapy versus fulvestrant or aromatase inhibitor monotherapy as second- or third-line therapy in patients with ER+/HER2− MBC previously treated with a CDK4/6 inhibitor. The emerging profile for palazestrant shows that it is unique among next-generation endocrine therapies in terms of monotherapy efficacy and combinability with CDK4/6 inhibitors, with lower incidence of class-specific AEs such as photopsia, bradycardia, and diarrhea with palazestrant monotherapy. Studies of palazestrant combined with palbociclib, ribociclib, alpelisib, and everolimus are ongoing and will further elucidate the potential of palazestrant as a novel endocrine therapy backbone for targeted combination approaches.

## Supplementary Information

Supplementary Material 1.

## Data Availability

Data from this clinical study can be made available to other researchers on request and approval by the study sponsor, subject to any appropriate data transfer agreements. Participant-level data are not available to share. Any data sharing request should be described in a research proposal and submitted to Olema Oncology. In line with current data privacy legislation, data transfer agreements do not allow disclosure of data to third parties.

## Abbreviations

AE: Adverse event
ALT: Alanine aminotransferase
AST: Aspartate aminotransferase
AUC_0-24_: Area under the concentration–time curve from 0 to 24 h
C_avg_: Average plasma concentration
CBR: Clinical benefit rate
CDK4/6: Cyclin-dependent kinase 4/6
CERAN: Complete estrogen receptor antagonist
cfDNA: Cell-free DNA
C_max_: Maximum plasma concentration
C_min_: Minimum plasma concentration
CNS: Central nervous system
cPR: Confirmed partial response
CR: Complete response
DLT: Dose-limiting toxicity
ECOG: Eastern Cooperative Oncology Group
ER: Estrogen receptor
ER +: Estrogen receptor–positive
*ESR1*: Estrogen receptor 1 gene
HER2 −: Human epidermal growth factor receptor 2–negative
MAF: Mutant allele frequency
MBC: Metastatic breast cancer
MTD: Maximum tolerated dose
PFS: Progression-free survival
PK: Pharmacokinetic(s)
PR: Partial response
QTcF: QT interval corrected by the Fridericia formula
RECIST v1.1: Response Evaluation Criteria in Solid Tumors, version 1.1
RP2D: Recommended phase 2 dose
SD: Stable disease
SERCA: Selective estrogen receptor covalent antagonist
SERD: Selective estrogen receptor degrader
SERM: Selective estrogen receptor modulator
t_1/2_: Effective half-life
TEAE: Treatment-emergent adverse event
T_max_: Time to maximum plasma concentration
TRAE: Treatment-related adverse event

## References

[1] Bray F, Laversanne M, Sung H, et al. Global cancer statistics 2022: GLOBOCAN estimates of incidence and mortality worldwide for 36 cancers in 185 countries. CA Cancer J Clin. 2024;74(3):229–63. 38572751 10.3322/caac.21834

[2] American Cancer Society. Breast Cancer Facts and Figures 2022-2024. Atlanta: American Cancer Society, Inc; 2022.

[3] Li Y, Yang D, Yin X, et al. Clinicopathological Characteristics and Breast Cancer-Specific Survival of Patients With Single Hormone Receptor-Positive Breast Cancer. JAMA Netw open. 2020;3(1):E1918160. 31899528 10.1001/jamanetworkopen.2019.18160 PMC6991239

[4] Giannakeas V. Single Hormone Receptor-Positive Breast Cancer—Signal or Noise? JAMA Netw Open. 2020;3(1):e1918176–e1918176. 31899524 10.1001/jamanetworkopen.2019.18176

[5] Hefti MM, Hu R, Knoblauch NW et al. Estrogen receptor negative/progesterone receptor positive breast cancer is not a reproducible subtype. Breast Cancer Res. 2013;15(4):R68. 10.1186/bcr3462 PMC3978610 23971947

[6] National Comprehensive Cancer Network (NCCN). NCCN Guidelines® Version 4.2025. Breast Cancer Guidelines. https://www.nccn.org/professionals/physician_gls/pdf/breast.pdf. Accessed 13 May 2025.

[7] Cardoso F, Di LA, Lohrisch C, Bernard C, Ferreira F, Piccart MJ. Second and subsequent lines of chemotherapy for metastatic breast cancer: what did we learn in the last two decades? Ann Oncol. 2002;13:197–207. 11885995 10.1093/annonc/mdf101

[8] Planchat E, Abrial C, Thivat E, et al. Late lines of treatment benefit survival in metastatic breast cancer in current practice? Breast. 2011;20(6):574–8. 21852136 10.1016/j.breast.2011.07.010

[9] Kaufman PA, Awada A, Twelves C, et al. Phase III open-label randomized study of eribulin mesylate versus capecitabine in patients with locally advanced or metastatic breast cancer previously treated with an anthracycline and a taxane. J Clin Oncol. 2015;33(6):594–601. 25605862 10.1200/JCO.2013.52.4892 PMC4463422

[10] Park M, Yu S-Y, Jeon H-L, Song I. Factors affecting health-related quality of life in patients with metastatic breast cancer. J Breast Cancer. 2023;26(5):436–45. 37565928 10.4048/jbc.2023.26.e29 PMC10625865

[11] Brett JO, Spring LM, Bardia A, Wander SA. ESR1 mutation as an emerging clinical biomarker in metastatic hormone receptor-positive breast cancer. Breast Cancer Res. 2021;23(1):85. 10.1186/s13058-021-01462-3 PMC8365900 34392831

[12] Rasha F, Sharma M, Pruitt K. Mechanisms of endocrine therapy resistance in breast cancer. Mol Cell Endocrinol. 2021;532:111322. 10.1016/j.mce.2021.111322 34000350

[13] Ma CX, Reinert T, Chmielewska I, Ellis MJ. Mechanisms of aromatase inhibitor resistance. Nat Rev Cancer. 2015;15(5):261–75. 25907219 10.1038/nrc3920

[14] Will M, Liang J, Metcalfe C, Chandarlapaty S. Therapeutic resistance to anti-oestrogen therapy in breast cancer. Nat Rev Cancer. 2023;23(10):673–85. 37500767 10.1038/s41568-023-00604-3 PMC10529099

[15] Parisian AD, Barratt SA, Hodges-Gallagher L, et al. Palazestrant (OP-1250), A Complete Estrogen Receptor Antagonist, Inhibits Wild-type and Mutant ER-positive Breast Cancer Models as Monotherapy and in Combination. Mol Cancer Ther. 2024;23(3):285–300. 38102750 10.1158/1535-7163.MCT-23-0351 PMC10911704

[16] Hodges-Gallagher L, Sun R, Myles DC, Harmon CL, Kushner PJ. OP-1250 is a complete estrogen receptor antagonist (CERAN) that lacks agonist activity on cell signaling and proliferation in breast cancer cells. Poster presented at: Poster presented at: The AACR Annual Meeting; 2020 Apr 27-28; virtual.

[17] Hodges-Gallagher L, Sun R, Myles DC, Harmon CL, Kushner PJ. Development of OP-1250, an oral complete estrogen receptor antagonist (CERAN) that shrinks ER-positive breast tumors in xenograft models. Poster presented at: The San Antonio Breast Cancer Symposium; 2019 Dec 10-14; San Antonio, TX, USA.

[18] Hodges-Gallagher L, Parisian AD, Sun R, Myles DC, Klein PM, Harmon CL, et al. The complete estrogen receptor antagonist (CERAN) OP-1250 shrinks ER+ breast cancer tumors expressing the ESR1-Y537S mutant estrogen receptor in an intracranial xenograft model of brain metastases. Poster presented at: The AACR Annual Meeting; 2021 April 9-14; virtual.

[19] Allison KH, Hammond MEH, Dowsett M, et al. Estrogen and Progesterone Receptor Testing in Breast Cancer: ASCO/CAP Guideline Update. J Clin Oncol. 2020;38(12):1346–66. 31928404 10.1200/JCO.19.02309

[20] Dowsett M, Nielsen TO, A’Hern R, et al. Assessment of Ki67 in breast cancer: recommendations from the International Ki67 in Breast Cancer working group. J Natl Cancer Inst. 2011;103(22):1656–64. 21960707 10.1093/jnci/djr393 PMC3216967

[21] Nielsen TO, Leung SCY, Rimm DL, et al. Assessment of Ki67 in Breast Cancer: Updated Recommendations From the International Ki67 in Breast Cancer Working Group. J Natl Cancer Inst. 2021;113(7):808–19. 33369635 10.1093/jnci/djaa201 PMC8487652

[22] Hamilton E, Meisel J, Alemany C, Borges V, Lin NU, Wesolowski R, et al. Preliminary phase 1/2 results from OP-1250-001, a study of OP-1250, an oral CERAN/SERD, in patients with advanced and/or metastatic estrogen receptor-positive, HER2-negative breast cancer (NCT04505826). Poster presented at: The EORTC-NCI-AACR Symposium on Molecular Targets and Cancer Therapeutics; 2022 Oct 26-28; Barcelona, Spain.

[23] Toy W, Shen Y, Won H, et al. ESR1 ligand-binding domain mutations in hormone-resistant breast cancer. Nat Genet. 2013;45(12):1439–45. 24185512 10.1038/ng.2822 PMC3903423

[24] Hamilton EP, Dees EC, Wang JS-Z et al. Phase I dose escalation of H3B-6545, a first-in-class highly Selective ERα Covalent Antagonist (SERCA), in women with ER-positive, HER2-negative breast cancer (HR+ BC). 2019;37(15_suppl):1059–1059. 10.1200/JCO.2019.37.15_suppl.1059.

[25] Hamilton EP, Oliveira M, Banerji U et al. A phase I dose escalation and expansion study of the next generation oral SERD AZD9833 in women with ER-positive, HER2-negative advanced breast cancer. 2020;38(15_suppl):1024–1024. 10.1200/JCO.2020.38.15_suppl.1024.

[26] Bardia A, Kaklamani V, Wilks S et al. Phase I study of elacestrant (RAD1901), a novel selective estrogen receptor degrader, in ER-positive, HER2-negative advanced breast cancer. 2021;39(12):1360–1370. 10.1200/JCO.20.02272. 10.1200/JCO.20.02272 PMC8078341 33513026

[27] Jhaveri K, Juric D, Yap YS, et al. A Phase I Study of LSZ102, an Oral Selective Estrogen Receptor Degrader, with or without Ribociclib or Alpelisib, in Patients with Estrogen Receptor-Positive Breast Cancer. Clin Cancer Res. 2021;27(21):5760. 34433648 10.1158/1078-0432.CCR-21-1095 PMC9401512

[28] Hamilton E, Oliveira M, Turner N, et al. A phase I dose escalation and expansion trial of the next-generation oral SERD camizestrant in women with ER-positive, HER2-negative advanced breast cancer: SERENA-1 monotherapy results. Ann Oncol. 2024;35(8):707–17. 38729567 10.1016/j.annonc.2024.04.012

[29] Jhaveri K, Winer EP, Lim E, et al. Abstract PD7–05: A first-in-human phase I study to evaluate the oral selective estrogen receptor degrader (SERD), GDC-9545, in postmenopausal women with estrogen receptor-positive (ER+) HER2-negative (HER2-) metastatic breast cancer. Cancer Res. 2020;80(4_Supplement):PD7-05.

[30] Cristofanilli M, Turner NC, Bondarenko I, et al. Fulvestrant plus palbociclib versus fulvestrant plus placebo for treatment of hormone-receptor-positive, HER2-negative metastatic breast cancer that progressed on previous endocrine therapy (PALOMA-3): final analysis of the multicentre, double-blind, phase 3 randomised controlled trial. Lancet Oncol. 2016;17(4):425–39. 26947331 10.1016/S1470-2045(15)00613-0

[31] Sledge GW, Toi M, Neven P, et al. MONARCH 2: Abemaciclib in Combination With Fulvestrant in Women With HR+/HER2- Advanced Breast Cancer Who Had Progressed While Receiving Endocrine Therapy. J Clin Oncol. 2017;35(25):2875–84. 28580882 10.1200/JCO.2017.73.7585

[32] Jhaveri KL, Neven P, Casalnuovo ML et al. Imlunestrant with or without Abemaciclib in Advanced Breast Cancer. N Engl J Med. 2025;392(12):1189-202.10.1056/NEJMoa241085839660834

[33] Bidard FC, Kaklamani VG, Neven P et al. Elacestrant (oral selective estrogen receptor degrader) versus standard endocrine therapy for estrogen receptor-positive, human epidermal growth factor receptor 2-negative advanced breast cancer: Results from the randomized phase III EMERALD trial. J Clin Oncol. 2022;40(28):3246-56. 10.1200/JCO.22.00338 PMC9553388 35584336

[34] Chan A, Day D, Dinh P, Slancar M, Lombard J, Ganju V, et al. A phase 1b/2 study of palazestrant (OP-1250), an oral complete estrogen receptor antagonist (CERAN) and selective ER degrader (SERD), with palbociclib in ER-positive, HER2-negative, advanced or metastatic breast cancer patients. Poster presented at: The San Antonio Breast Cancer Symposium; 2023 Dec 5-9; San Antonio, TX, USA.

[35] Borges V, Alemany C, Lin N, Nunnery S, Ma C, Tonda M, et al. A phase 1b/2 study of palazestrant (OP-1250) in combination with ribociclib in patients with estrogen receptor-positive, human epidermal growth factor receptor 2-negative, advanced and/or metastatic breast cancer. Poster presented at: The San Antonio Breast Cancer Symposium. 2024 Dec 10-13; San Antonio, TX, USA.

[36] Borges VF, Chien J, Nunnery S, Mouabbi JA, Ma CX, Bahadur S, et al. A phase Ib/II study of palazestrant (OP-1250) in combination with ribociclib in patients with estrogen receptor-positive human epidermal growth factor receptor 2-negative (ER+, HER2-) metastatic breast cancer. Poster presented at: ESMO Breast Cancer Annual Congress. 2024 May 15-17; Berlin.

[37] Kisqali [package insert on the Internet]. East Hanover (NJ): Novartis Pharmaceuticals Corporation; 2017 [updated 2025 Feb; cited 2025 May 13]. Available from: https://www.novartis.com/us-en/sites/novartis_us/files/kisqali.pdf.

[38] Ibrance [package insert on the Internet]. New York (NY): Pfizer Inc.; 2015 [updated 2025 Apr; cited 2025 May 13]. Available from: https://labeling.pfizer.com/ShowLabeling.aspx?id=2191.

[39] Slamon DJ, Neven P, Chia S, et al. Overall Survival with Ribociclib plus Fulvestrant in Advanced Breast Cancer. N Engl J Med. 2020;382(6):514–24. 31826360 10.1056/NEJMoa1911149

[40] Turner NC, Ro J, André F et al. Palbociclib in hormone-receptor–positive advanced breast cancer. 2015. 10.1056/NEJMc1510345 26488700

[41] Lin NU, Borges VF, Patel MR, Okera M, Meisel J, Wesolowski R, et al. Results from the phase I/II study of OP-1250, an oral complete estrogen receptor (ER) antagonist (CERAN) and selective ER degrader (SERD) in patients (pts) with advanced or metastatic ER-positive, HER2-negative breast cancer. Oral abstractpresented at: ESMO Congress; 2023 Oct 20-24; Madrid, Spain.

[42] Thill M, Kolberg-Liedtke C, Albert US, et al. AGO Recommendations for the diagnosis and treatment of patients with locally advanced and metastatic breast cancer: Update 2023. Breast Care (Basel). 2023;18(4):305–14. 10.1159/000531579 PMC10601669 37900553

[43] Jhaveri KL, Lim E, Jeselsohn R, et al. Imlunestrant, an Oral Selective Estrogen Receptor Degrader, as Monotherapy and in Combination With Targeted Therapy in Estrogen Receptor-Positive, Human Epidermal Growth Factor Receptor 2–Negative Advanced Breast Cancer: Phase Ia/Ib EMBER Study. J Clin Oncol. 2024;42(35):4173. 39241211 10.1200/JCO.23.02733 PMC11637582

[44] Wardell SE, Nelson ER, Chao CA, Alley HM, McDonnell DP. Evaluation of the pharmacological activities of RAD1901, a selective estrogen receptor degrader. Endocr Relat Cancer. 2015;22(5):713–24. 26162914 10.1530/ERC-15-0287 PMC4545300

[45] Ferreira Almeida C, Oliveira A, João Ramos M, Fernandes PA, Teixeira N, Amaral C. Estrogen receptor-positive (ER+) breast cancer treatment: Are multi-target compounds the next promising approach? Biochem Pharmacol. 2020;177:113989. 32330493 10.1016/j.bcp.2020.113989

[46] Guan J, Zhou W, Hafner M, et al. Therapeutic ligands antagonize estrogen receptor function by impairing its mobility. Cell. 2019;178(4):949-963.e18. 31353221 10.1016/j.cell.2019.06.026

