# Supplementary material for: Palazestrant, a novel oral Complete Estrogen Receptor Antagonist (CERAN) and Selective Estrogen Receptor Degrader (SERD), in patients with ER+/HER2− advanced or metastatic breast cancer: phase 1/2 study results
Source: Breast Cancer Res. 2025 Jul 1;27:119. doi: 10.1186/s13058-025-02049-y (PMC12210654; doi:10.1186/s13058-025-02049-y)
Supplement: Supplementary file 1 — Supplementary Material 1. [file 13058_2025_2049_MOESM1_ESM.pdf]

Supplemental Figure 1. Duration of treatment and best overall responses

Duration of treatment and best overall responses in patients in the dose-escalation cohorts (phase 1a). Each lane represents one patient. Solid purple boxes represent *ESR1* mutation, solid pink boxes represent *ESR1* wild-type, and the absence of a box indicates missing data. *Progression by radiographic assessment only.

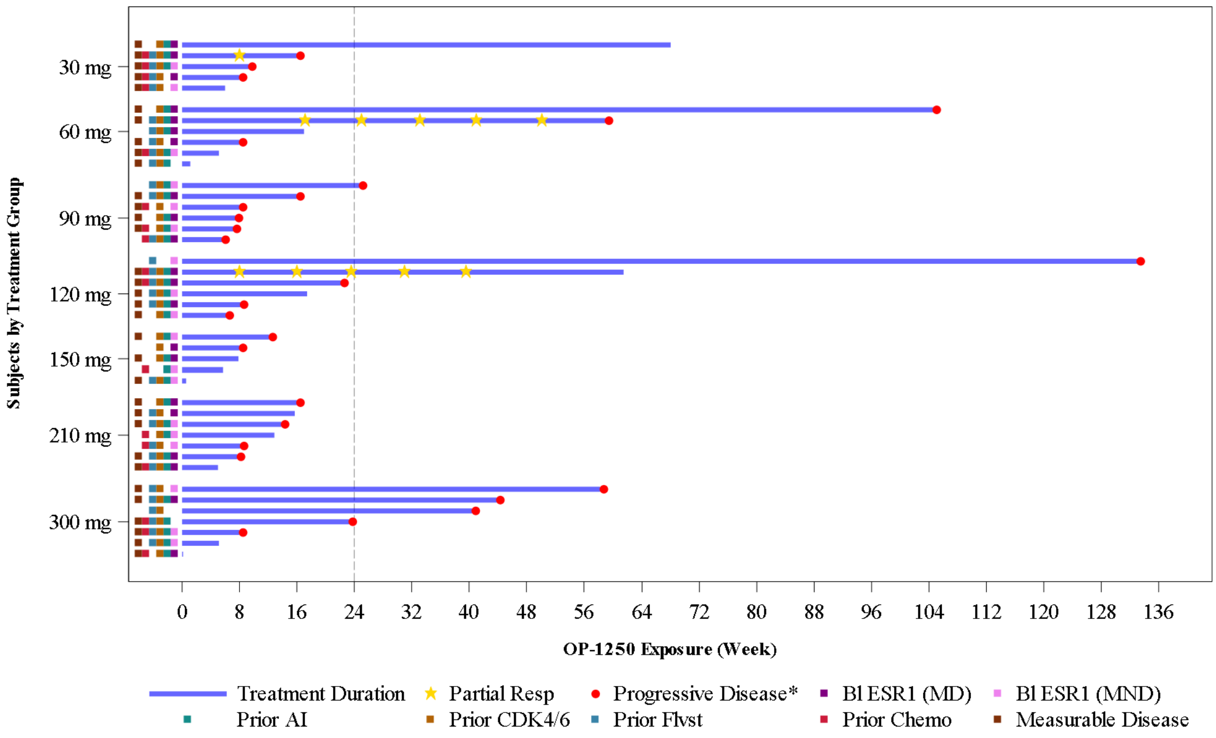

AI, aromatase inhibitor; Bl, baseline; CDK4/6i, cyclin-dependent kinase 4/6 inhibitor; Chemo, chemotherapy; *ESR1*, estrogen receptor 1; Fulv, fulvestrant, MD, mutation detected, MND, mutation not detected.

Supplemental Figure 2. Biomarker analyses following treatment with palazestrant.

Quantitative analyses were performed on patient tissue and blood samples collected at baseline and cycle 3 day 1 of palazestrant treatment. A) The percentage of Ki67-posive nuclei was significantly decreased after treatment with palazestrant compared (*P*=.041). B) ER Allred scores were decreased after treatment with palazestrant (*P*=.13). C) Sankey diagram showing the number of mutations detected in cfDNA for each *ESR1* variant before and after treatment with palazestrant. N = number of patients in the analysis; n= number of mutations. Out of 64 patients who had an *ESR1* mutation detected at C1D1, 49 (77%) had at least one mutation become “Not detected” at C3D1. Out of 64 patients who had an *ESR1* mutation detected at C1D1, 43 (67%) had all their detected mutation(s) become “Not detected” at C3D1.

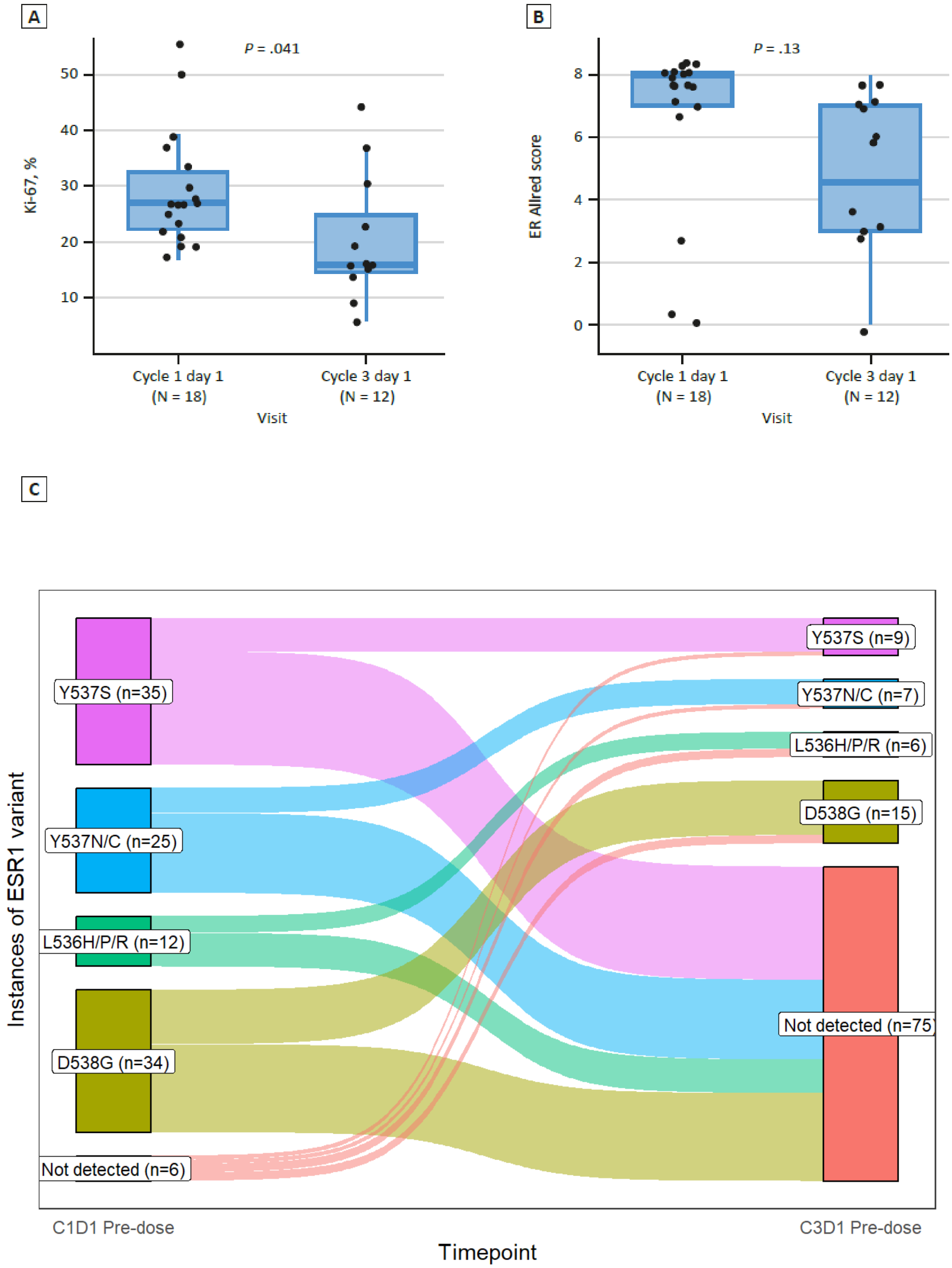

cfDNA, cell free DNA; ER, estrogen receptor; ESR1, estrogen receptor 1 gene.

27 **Supplementary Table 1. Patient baseline characteristics and demographics (dose escalation cohort; phase 1a)**

		Treatment							
		30 mg QD (N=5)	60 mg QD (N=6)	90 mg QD (N=6)	120 mg QD (N=6)	150 mg QD (N=5)	210 mg QD (N=7)	300 mg QD (N=7)	Total (N=42)
Age* (year)	n	5	6	6	6	5	7	7	42
	Mean (SD)	62.0 (7.58)	62.2 (15.57)	54.2 (7.03)	57.8 (10.61)	70.8 (17.91)	67.7 (8.24)	63.9 (14.58)	62.6 (12.41)
	Median	63.0	65.0	57.0	62.5	76.0	65.0	64.0	63.5
	Min, Max	54, 72	34, 79	40, 58	40, 67	40, 85	57, 77	37, 82	34, 85
Age Group*	< 65	3 (60.0)	3 (50.0)	6 (100)	4 (66.7)	1 (20.0)	3 (42.9)	4 (57.1)	24 (57.1)
	65 - <75	2 (40.0)	2 (33.3)	0 (0.0)	2 (33.3)	1 (20.0)	2 (28.6)	1 (14.3)	10 (23.8)
	≥ 75	0 (0.0)	1 (16.7)	0 (0.0)	0 (0.0)	3 (60.0)	2 (28.6)	2 (28.6)	8 (19.0)
Sex	Male	0 (0.0)	0 (0.0)	0 (0.0)	0 (0.0)	0 (0.0)	1 (14.3)	0 (0.0)	1 (2.4)
	Female	5 (100)	6 (100)	6 (100)	6 (100)	5 (100)	6 (85.7)	7 (100)	41 (97.6)
Race	Black or African American	0 (0.0)	0 (0.0)	0 (0.0)	1 (16.7)	0 (0.0)	0 (0.0)	0 (0.0)	1 (2.4)
	White	5 (100)	5 (83.3)	4 (66.7)	5 (83.3)	5 (100)	7 (100)	6 (85.7)	37 (88.1)
	Not Reported	0 (0.0)	1 (16.7)	2 (33.3)	0 (0.0)	0 (0.0)	0 (0.0)	1 (14.3)	4 (9.5)
Menopausal Status (Female)	Post-menopausal	5 (100)	6 (100)	6 (100)	6 (100)	4 (80.0)	6 (100)	7 (100)	40 (97.6)
	Peri/pre-menopausal and taking an LHRH agonist	0 (0.0)	0 (0.0)	0 (0.0)	0 (0.0)	1 (20.0)	0 (0.0)	0 (0.0)	1 (2.4)
Baseline ECOG Score	0	2 (40.0)	5 (83.3)	1 (16.7)	1 (16.7)	1 (20.0)	2 (28.6)	6 (85.7)	18 (42.9)
	1	3 (60.0)	1 (16.7)	5 (83.3)	5 (83.3)	4 (80.0)	5 (71.4)	1 (14.3)	24 (57.1)
Presence of Visceral Disease (Liver, Lung, Pleura, Ascites, or Peritoneum)	Yes	3 (60.0)	4 (66.7)	2 (33.3)	5 (83.3)	2 (40.0)	4 (57.1)	5 (71.4)	25 (59.5)
	No	2 (40.0)	2 (33.3)	4 (66.7)	1 (16.7)	3 (60.0)	3 (42.9)	2 (28.6)	17 (40.5)
Prior Lines of Therapy in Advanced Setting		5 (100)	6 (100)	6 (100)	6 (100)	5 (100)	7 (100)	7 (100)	42 (100)
	1	1 (20.0)	2 (33.3)	1 (16.7)	1 (16.7)	2 (40.0)	0 (0.0)	2 (28.6)	9 (21.4)
	2	1 (20.0)	2 (33.3)	2 (33.3)	2 (33.3)	2 (40.0)	3 (42.9)	0 (0.0)	12 (28.6)
	3	0 (0.0)	0 (0.0)	2 (33.3)	1 (16.7)	1 (20.0)	2 (28.6)	2 (28.6)	8 (19.0)

		Treatment							
		30 mg QD (N=5)	60 mg QD (N=6)	90 mg QD (N=6)	120 mg QD (N=6)	150 mg QD (N=5)	210 mg QD (N=7)	300 mg QD (N=7)	Total (N=42)
	4	1 (20.0)	1 (16.7)	1 (16.7)	0 (0.0)	0 (0.0)	1 (14.3)	1 (14.3)	5 (11.9)
	5 or More	2 (40.0)	1 (16.7)	0 (0.0)	2 (33.3)	0 (0.0)	1 (14.3)	2 (28.6)	8 (19.0)
	n	5	6	6	6	5	7	7	42
	Mean (SD)	3.6 (2.07)	3.0 (2.68)	2.5 (1.05)	3.2 (1.94)	1.8 (0.84)	3.0 (1.15)	3.3 (1.89)	2.9 (1.72)
	Median	4.0	2.0	2.5	2.5	2.0	3.0	3.0	2.5
	Min, Max	1, 6	1, 8	1, 4	1, 6	1, 3	2, 5	1, 6	1, 8
Prior Lines of Endocrine Therapy in Advanced Setting [AI/Fulv/Inv/LHRH/SERM]		5 (100)	6 (100)	6 (100)	6 (100)	5 (100)	7 (100)	7 (100)	42 (100)
	1	2 (40.0)	2 (33.3)	3 (50.0)	1 (16.7)	3 (60.0)	1 (14.3)	2 (28.6)	14 (33.3)
	2	1 (20.0)	2 (33.3)	2 (33.3)	2 (33.3)	1 (20.0)	3 (42.9)	2 (28.6)	13 (31.0)
	3	1 (20.0)	0 (0.0)	0 (0.0)	2 (33.3)	1 (20.0)	2 (28.6)	1 (14.3)	7 (16.7)
	4	0 (0.0)	1 (16.7)	1 (16.7)	0 (0.0)	0 (0.0)	1 (14.3)	2 (28.6)	5 (11.9)
	5 or More	1 (20.0)	1 (16.7)	0 (0.0)	1 (16.7)	0 (0.0)	0 (0.0)	0 (0.0)	3 (7.1)
	n	5	6	6	6	5	7	7	42
	Mean (SD)	2.4 (1.67)	2.5 (1.64)	1.8 (1.17)	2.7 (1.37)	1.6 (0.89)	2.4 (0.98)	2.4 (1.27)	2.3 (1.25)
	Median	2.0	2.0	1.5	2.5	1.0	2.0	2.0	2.0
	Min, Max	1, 5	1, 5	1, 4	1, 5	1, 3	1, 4	1, 4	1, 5
Prior Types of Anti-Cancer Treatment in Advanced Setting		5 (100)	6 (100)	6 (100)	6 (100)	5 (100)	7 (100)	7 (100)	42 (100)
[- Chemo]	Chemotherapy	4 (80.0)	1 (16.7)	3 (50.0)	2 (33.3)	1 (20.0)	3 (42.9)	3 (42.9)	17 (40.5)
[- Endocrine]	Aromatase Inhibitor (AI)	3 (60.0)	5 (83.3)	5 (83.3)	5 (83.3)	4 (80.0)	5 (71.4)	5 (71.4)	32 (76.2)
	Fulvestrant	4 (80.0)	5 (83.3)	3 (50.0)	5 (83.3)	1 (20.0)	5 (71.4)	6 (85.7)	29 (69.0)
	Investigational Endocrine	0 (0.0)	1 (16.7)	0 (0.0)	0 (0.0)	0 (0.0)	1 (14.3)	0 (0.0)	2 (4.8)
	LHRH Agonist	0 (0.0)	0 (0.0)	2 (33.3)	2 (33.3)	0 (0.0)	1 (14.3)	0 (0.0)	5 (11.9)
	SERM	2 (40.0)	0 (0.0)	2 (33.3)	1 (16.7)	0 (0.0)	0 (0.0)	1 (14.3)	6 (14.3)
[- Targeted]	CDK4/6 Inhibitor	5 (100)	6 (100)	6 (100)	5 (83.3)	4 (80.0)	7 (100)	7 (100)	40 (95.2)
	Investigational Agent	0 (0.0)	0 (0.0)	0 (0.0)	2 (33.3)	0 (0.0)	1 (14.3)	0 (0.0)	3 (7.1)
	Other Targeted	1 (20.0)	1 (16.7)	0 (0.0)	0 (0.0)	0 (0.0)	1 (14.3)	2 (28.6)	5 (11.9)
	PI3K Inhibitor	1 (20.0)	1 (16.7)	2 (33.3)	1 (16.7)	0 (0.0)	0 (0.0)	0 (0.0)	5 (11.9)
	mTOR Inhibitor	1 (20.0)	2 (33.3)	2 (33.3)	1 (16.7)	1 (20.0)	2 (28.6)	3 (42.9)	12 (28.6)
	n	5	6	6	6	5	7	7	42

		Treatment							
		30 mg QD (N=5)	60 mg QD (N=6)	90 mg QD (N=6)	120 mg QD (N=6)	150 mg QD (N=5)	210 mg QD (N=7)	300 mg QD (N=7)	Total (N=42)
	Mean (SD)	4.4 (1.82)	3.7 (2.25)	4.3 (1.86)	4.3 (1.63)	2.6 (0.89)	4.1 (1.77)	3.9 (1.46)	3.9 (1.69)
	Median	4.0	3.0	4.0	4.5	2.0	4.0	4.0	4.0
	Min, Max	2, 7	2, 8	2, 7	2, 6	2, 4	3, 8	2, 6	2, 8
ESR1 Mutations at Baseline(ctDNA)	Patients with C1D1 Evaluation	5	5	6	6	5	7	5	39
	Mutation Detected	3 (60.0)	4 (80.0)	3 (50.0)	3 (50.0)	2 (40.0)	4 (57.1)	2 (40.0)	21 (53.8)
	Mutation not Detected	2 (40.0)	1 (20.0)	3 (50.0)	3 (50.0)	3 (60.0)	3 (42.9)	3 (60.0)	18 (46.2)
Measurable Disease		5 (100)	5 (83.3)	4 (66.7)	5 (83.3)	3 (60.0)	5 (71.4)	6 (85.7)	33 (78.6)

Supplemental Table 2. TEAEs Occurring in ≥15% of Patients Overall (Dose-Escalation Cohort; Phase 1a)

Preferred Term	Toxicity Grade	30 mg (N=5)	60 mg (N=6)	90 mg (N=6)	120 mg (N=6)	150 mg (N=5)	210 mg (N=7)	300 mg (N=7)	Total (N=42)
Nausea	All	2 (40.0)	2 (33.3)	2 (33.3)	5 (83.3)	4 (80.0)	4 (57.1)	6 (85.7)	25 (59.5)
	G3	0	0	0	0	1 (20.0)	0	1 (14.3)	2 (4.8)
	G4	0	0	0	0	0	0	0	0
Fatigue	All	2 (40.0)	3 (50.0)	0	2 (33.3)	1 (20.0)	2 (28.6)	4 (57.1)	14 (33.3)
	G3	0	0	0	0	1 (20.0)	0	0	1 (2.4)
	G4	0	0	0	0	0	0	0	0
Vomiting	All	1 (20.0)	1 (16.7)	1 (16.7)	2 (33.3)	2 (40.0)	1 (14.3)	5 (71.4)	13 (31.0)
	G3	0	0	0	1 (16.7)	0	0	0	1 (2.4)
	G4	0	0	0	0	0	0	0	0
Constipation	All	1 (20.0)	1 (16.7)	1 (16.7)	0	0	4 (57.1)	3 (42.9)	10 (23.8)
	G3	0	0	0	0	0	0	0	0
	G4	0	0	0	0	0	0	0	0
Decreased appetite	All	1 (20.0)	0	1 (16.7)	1 (16.7)	1 (20.0)	3 (42.9)	3 (42.9)	10 (23.8)
	G3	0	0	0	0	0	0	0	0
	G4	0	0	0	0	0	0	0	0
Headache	All	1 (20.0)	1 (16.7)	1 (16.7)	1 (16.7)	1 (20.0)	0	4 (57.1)	9 (21.4)
	G3	0	0	0	0	0	0	0	0
	G4	0	0	0	0	0	0	0	0
Neutropenia[a]	All	1 (20.0)	1 (16.7)	0	2 (33.3)	2 (40.0)	1 (14.3)	3 (42.9)	10 (23.8)
	G3	0	0	0	0	0	0	0	0
	G4	1 (20.0)	0	0	1 (16.7)	1 (20.0)	0	1 (14.3)	4 (9.5)
Diarrhea	All	2 (40.0)	2 (33.3)	0	1 (16.7)	1 (20.0)	0	1 (14.3)	7 (16.7)
	G3	0	0	0	0	0	0	0	0
	G4	0	0	0	0	0	0	0	0
Maculopapular rash	All	1 (20.0)	1 (16.7)	0	1 (16.7)	0	2 (28.6)	2 (28.6)	7 (16.7)
	G3	0	0	0	0	0	0	0	0
	G4	0	0	0	0	0	0	0	0

[a]Includes preferred terms "neutropenia" and "neutrophil count decreased." TEAE, treatment-emergent adverse event.
